# Sex Differences and the Impact of Chronic Stress and Recovery on Instrumental Learning

**DOI:** 10.1155/2015/697659

**Published:** 2015-04-16

**Authors:** Angela L. McDowell, Kathryn M. H. Fransen, Kevin S. Elliott, Alhasan Elghouche, Polina V. Kostylev, Pamela K. O'Dea, Preston E. Garraghty

**Affiliations:** ^1^Department of Psychological and Brain Sciences, Indiana University, Bloomington, IN 47405, USA; ^2^Program in Neuroscience, Indiana University, Bloomington, IN 47405, USA

## Abstract

We have previously shown that 21-day chronic restraint stress impacts instrumental learning, but overall few studies have examined sex differences on the impact of stress on learning. We further examined sex differences in response to extended 42-day chronic stress on instrumental learning, as well as recovery from chronic stress. Rats were tested in aversive training tasks with or without prior appetitive experience, and daily body weight data was collected as an index of stress. Relative to control animals, reduced body weight was maintained from day 22 through day 42 across the stress period for males, but not for females. Stressed males had increased response speed and lower learning efficiency during appetitive acquisition and aversive learning. Males overall showed slower escape shaping times and more shock exposure. In contrast, stressed females showed slower appetitive response speeds and higher appetitive and aversive efficiency but overall reduced avoidance rates during acquisition and maintenance for transfer animals and during maintenance for aversive-only animals. These tasks reveal important nuances on the effect of stress on goal-directed behavior and further highlight sexually divergent effects on appetitive versus aversive motivation. Furthermore, these data underscore that systems are temporally impacted by chronic stress in a sexually divergent pattern.

## 1. Introduction

Chronic stress can lead to learning deficits in humans [1] and has been shown to be moderated by sex differences [2–4]. Rodent models of chronic stress have been developed to study the impact of chronic stress on morphological changes in the brain and learning [5–7]. More recently the role of sex differences and the response to chronic stress have also been investigated [8–10]. Studies show that interactions between stress hormones and gonadal hormones differentially impact learning and motivation centers in the brain leading to sexually dimorphic responses [10–12].

We have previously used the instrumental appetitive-to-aversive transfer task to investigate a variety of manipulations [13–17] on learning outcomes. Advantages of using these instrumental tasks are the ability to test lower cognitive function (i.e., shaping and nontransfer signaled learning) and directly compare the results to higher level cognitive function (i.e., appetitive-to-aversive transfer learning) while utilizing similar task demands. Furthermore, one study from our lab suggested that these instrumental tasks were good paradigms to reveal important nuances of sex-related hormones [17].

In a subsequent study we specifically investigated the impact of 21-day chronic restraint stress and sex differences on the appetitive-to-aversive transfer task and found task by sex by stress specific deficits [18]. While all males were impaired in escape shaping and stressed males were delayed in reaching asymptotic performance in transfer avoidance training, stressed females were facilitated in appetitive learning and showed modest deficits in transfer avoidance percentage. Although few studies have examined morphological changes in response to 21-day restraint stress [5, 19–21], only a couple studies have looked at changes in brain morphology in animals with extended restraint duration from day 21 through day 42 suggesting that this extended time may be neurologically and potentially clinically relevant [7, 21].

The current study is a follow-up to our previous publication and examines several questions. Are there impairments with 42 days of chronic restraint stress? Can male and female rats recover from the impact of 42 days of chronic restraint stress? We hypothesized that the extended duration of six weeks of chronic restraint stress would produce learning deficits relative to control animals and that the avoidance learning deficits would be exacerbated relative to those previously seen with three-week chronic restraint stress for males and females. Finally, we hypothesized that both males and females that were restrained for 42 days would recover with three weeks of nonrestraint prior to training.

## 2. Materials and Methods

### 2.1. Subjects

Adult Sprague-Dawleyrats weighing between 175 and 275 g (females) and 275 and 375 g (males) were randomly assigned to one of twelve groups. There were three experimental conditions (control, restraint, or recovery), two learning tasks (appetitive-to-aversive transfer or aversive-only), and two sexes (male or female). Animals were housed socially until experiment onset. Due to food restriction for the appetitive training, all animals were housed individually five days prior to experiment onset and were maintained at 85% of their free-feeding weight throughout all phases of instrumental training. Weighing occurred immediately prior to training and subsequently the animal was returned to its home when food availability began. Food restriction began five days before the first day of shaping onset for all animals. The rats had access to water* ad libitum* in their home cage and were housed in a 12:12 hour lighting environment with light on beginning at 7 a.m. All testing and manipulations occurred during light on. Some of the animals were tested in the appetitive-to-aversive transfer task (AP-AV): control males (denoting data that were previously published in a “sister” publication (McDowell et al., 2013)), restrained males, recovered males, control females (denoting data that were previously published in a “sister” publication (McDowell et al., 2013)), restrained females, and recovered females. The remaining animals were tested in the aversive-only task without prior appetitive training (AV): control males (denoting data that were previously published in a “sister” publication (McDowell et al., 2013)), restrained males, recovered males, control females (denoting data that were previously published in a “sister” publication (McDowell et al., 2013)), restrained females, and recovered females. All procedures were done ethically and approved by the Indiana University Institutional Animal Care and Use Committee (IACUC).

### 2.2. Stress Procedures

#### 2.2.1. Chronic Restraint

Animals were transported to a separate room and restrained for six hours per day between the hours of 10 a.m. to 4 p.m., seven days per week, for 42 consecutive days as previously described in detail [18]. All restrained rats were removed from their home cages and taken to a separate room during the restraining time period and were returned following restraint. The nonrestrained control animals were handled daily.

#### 2.2.2. Recovery from Stress

To examine the impact of recovery, a group of animals underwent the same 42-day chronic restraint protocol, but testing did not occur immediately following the last day of restraint. Instead rats were given three weeks of additional “recovery” time to sit in their home cages prior to beginning appetitive or escape shaping. Depending upon whether the animal was in the transfer group or the aversive-only group, appetitive or aversive shaping began the day immediately after the 21-day recovery period.

### 2.3. Littermate Controls

Separate male and female litter-matched animals underwent the same experimental restraint procedures to assess body weight changes (the first 21 days of these data were published in [18]). Animals in both groups (*n* = 22) were weighed daily as whole body weight has been shown to provide a valid index of stress [22].

### 2.4. Learning Procedures

#### 2.4.1. Appetitive Paradigm (AP)

Animals were appetitively shaped using a method of successive approximations to barpress for food reward (sugar pellet), which began one day immediately following the last day of restraint or recovery. Beginning on a fixed ratio (FR-1) schedule, when the animal barpressed 100 times within 30 minutes it was advanced to a fixed ratio-4 (FR-4) schedule of reinforcement. On the FR-4 schedule animals had to barpress 400 times to receive 100 food rewards within 40 min for two consecutive days in order to advance. After meeting the FR-4 schedule animals advanced to appetitive tone training in which a 3 sec tone (2000 Hz, 90 dB SPL) signaled the availability of food reward if the bar was pressed within the 3 sec tone period. One tone-signaled training session consisted of 100 trials (for details see [18]). Animals remained in appetitive training until it achieved 90% correct response (CR) rate or higher (i.e., barpressing during the tone) for two consecutive days. After the second day of meeting the appetitive criterion the animal advanced (transferred) to aversive learning to begin escape shaping which occurred in the same learning chamber.

#### 2.4.2. Aversive Paradigm (AV)

On the day immediately following appetitive training transfer animals began the aversive learning task. Additional groups were placed directly into the escape shaping task with no prior appetitive experience. In order to directly compare the impact of chronic restraint between transfer and AV-only animals, the average number of days transfer animals spent in the appetitive paradigm was calculated. AV-only animals waited the average number of days after restraint prior to beginning escape shaping. The aversive shaping program consisted of a shock that could be escaped with a lever press. The shock intensity was maintained between 0.8 and 1.0 mA and the shock pulses (250 ms at 1.33 Hz) were presented continuously until the bar was pressed. If the animal did not press the bar within 30 pulses, the current was turned off manually for a rest period of 10 seconds; however, when the animal barpressed, a rest period of 30 seconds was initiated. The escape shaping criterion was a barpress within 5 shock pulses or fewer for 15 consecutive escape trials. After meeting the escape criterion, animals were then advanced to tone-signaled (same as above) trials on the subsequent training day. On tone-signaled trials, four foot shock pulses of 250 ms separated by 500 ms could be avoided with a barpress during the first 3 seconds of tone presentation or escaped by a barpress in the latter 3 seconds after the shock pulses began. To prevent the animals from adopting a strategy of constantly holding the bar down to avoid shock, shock pulses (off-bar shocks, OBSs) were delivered after 5 seconds of holding and continued until the animal released the bar (for details see [18]). There were a total of ten training sessions and one session of tone-signaled avoidance training consisted of 300 trials or a maximum of two hours (whichever occurred first). Learning acquisition days were defined as training day one up to the day that preceded asymptotic performance. The learning asymptote was defined as the number of days it took for each animal to reach its own median avoidance rate across all ten days.

### 2.5. Statistical Analysis

Data are presented as mean ± standard error of the mean (SEM). A condition (3) × sex (2) analysis of variance (ANOVA) model was used to test all appetitive shaping data, all appetitive tone-signaled data, aversive efficiency ratios (ERs), aversive off-bar shocks (OBSs), and aversive difference scores. A condition (3) × sex (2) × task (2) ANOVA model was used to test escape shaping (number of days to criterion) data. Pearson's chi-square analysis was used to analyze group differences in the ability to meet the escape shaping criteria. A condition (2) × time (21) repeated measure analysis of variance (RM-ANOVA) test was used to evaluate body weight data for males and females and a condition (3) × sex (2) × days (10) RM-ANOVA was used to test avoidance learning. When assumptions of sphericity were violated, the degrees of freedom were adjusted using the Huynh-Feldt correction. To determine what was driving any overall significant effects, post hoc tests (more than two groups) or pairwise comparisons that were significant were reported. Additionally, repeated contrasts looked at learning improvement via day-by-day comparisons for all repeated measures. Multiple comparisons were adjusted using the Bonferroni correction and statistical significance was set at *p* ≤ 0.05. Asymptotic performance during avoidance learning was evaluated by calculating the number of days it took for each animal to reach its own median avoidance response rate and then averaging the within group rates for comparison across groups.

## 3. Results

### 3.1. Body Weight Assessment

**Figure 1:**
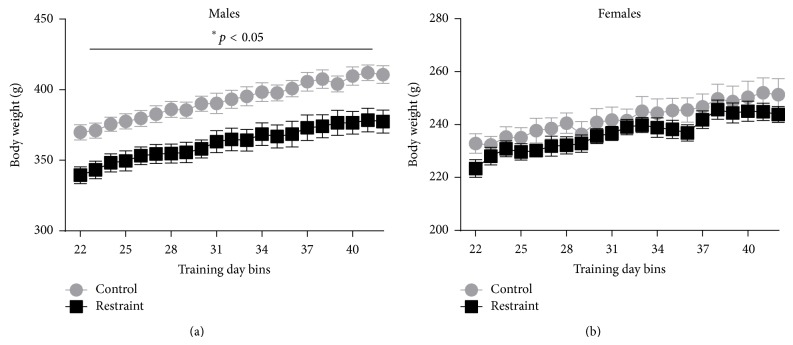
It shows body weight for males (a) and females (b) from training day 22 through day 42. Stressed males (black) gained weight at an attenuated rate (−1.2%) relative to controls (gray), whereas females gained weight at comparable rates.

Figure 1 shows data from body weight assessments from the separate group of litter-matched animals. The first 21 days of restraint for these animals were published [18], which found a significant impact of 21 days of chronic restraint stress on male and female animals across that time period. Animals were assigned to either chronic restraint (female, *n* = 5; male, *n* = 5) or to a control group (female, *n* = 6; male, *n* = 6) and restraint days 22 through 42 are shown in Figure 1. The average % weight gain for males (a), between restraint days 22 and 42, was 5.2% for controls and 4.0% for restrained animals, whereas the average gain in weight for females (b) was 2.7% for controls and 2.6% for restrained animals. The results revealed an overall significant main effect of sex [*F*_(1,18)_ = 622.961, *p* < 0.01], a main effect of condition [*F*_(1,18)_ = 4.868, *p* < 0.01], and an interaction of sex by condition [*F*_(1,18)_ = 4.868, *p* < 0.05], but there was no significant interaction of sex by condition across days [*F*_(20,360)_ = 0.885, *p* > 0.05].

### 3.2. Appetitive Learning

#### 3.2.1. Appetitive Shaping

**Figure 2:**
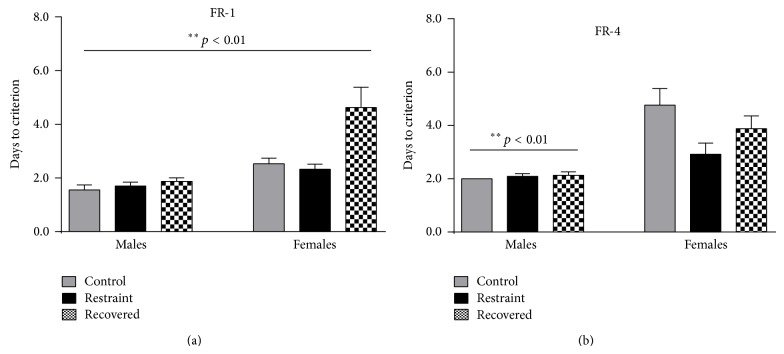
It shows appetitive shaping data for the fixed ratio-1 task (a) and the fixed ratio-4 task (b) for control (gray), stressed (black), and recovered (checkered) animals. There was significant sex by condition interaction on the FR-1 task with females and recovered animals taking longer to meet the criterions. There was a main effect of sex on the FR-4 task with females taking longer to meet the appetitive shaping criterion.

Figure 2 presents the average number of days it took for males and females to reach the appetitive shaping criterions for FR-1 (a) and FR-4 (b). The average number of days it took for males to reach the FR-1 criterion was 1.6 ± 0.2 (controls, *n* = 9), 1.7 ± 0.2 (restrained, *n* = 10), and 1.9 ± 0.1 (recovered, *n* = 8) and the FR-4 criterion was 2.0 ± 0.0 (controls), 2.1 ± 0.1 (restrained), and 2.3 ± 0.1 (recovered). The average number of days it took for females to reach the FR-1 criterion was 2.5 ± 0.2 (control, *n* = 17), 2.3 ± 0.2 (restrained, *n* = 12), and 4.6 ± 0.8 (recovered, *n* = 8) and the FR-4 criterion was 4.8 ± 0.6 (control), 2.9 ± 0.4 (restrained), and 3.9 ± 0.5 (recovered). There was an overall significant main effect for sex [*F*_(1,63)_ = 33.818, *p* < 0.01] and condition [*F*_(2,63)_ = 9.462, *p* < 0.01] as well as an interaction of sex by condition for the FR-1 task [*F*_(2,63)_ = 6.155, *p* < 0.01], which was accounted for by the significant difference between control and recovered (*p* < 0.01) and restraint and recovered animals (*p* < 0.01). On the FR-4 task there was a significant effect of sex [*F*_(1,63)_ = 18.637, *p* < 0.01], but no significant effect of condition [*F*_(2,63)_ = 1.714, *p* > 0.05] or interaction [*F*_(2,63)_ = 2.128, *p* > 0.05]. Males were faster at meeting the FR-4 criterion. All male and female rats learned to barpress for food reward during the appetitive shaping tasks and advanced to tone-signaled training.

#### 3.2.2. Appetitive Tone-Signaled Learning

**Figure 3:**
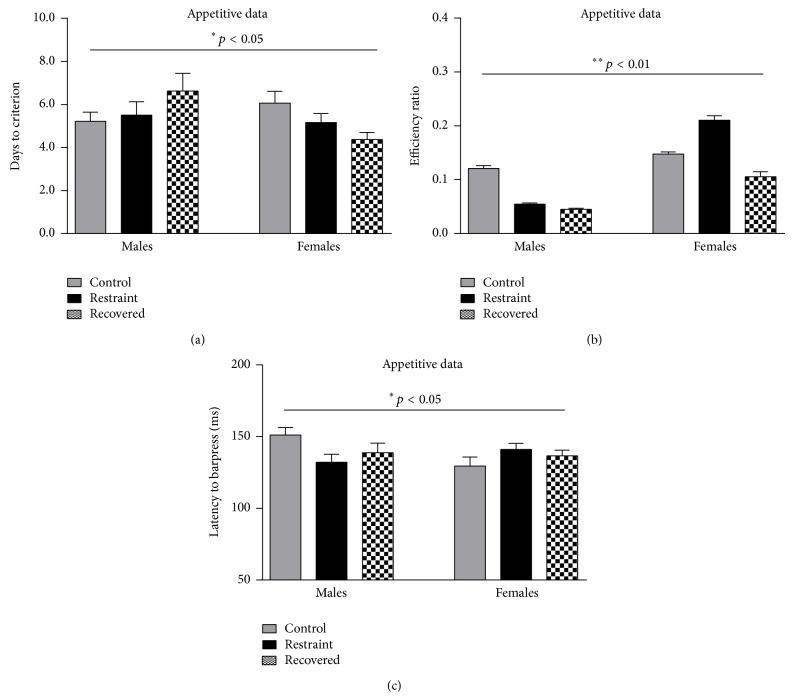
It shows appetitive tone-signaled data for the number of days to criterion (a), efficiency ratios during acquisition (b), and the latency to barpress during acquisition (c) for control (gray), stressed (black), and recovered (checkered) animals. There was a significant sex by condition interaction on all three measures. Whereas chronic stress reduced learning and increased response speed in males, it increased learning and reduced response speed in females. Recovery from stress showed variable results across measures.

Figure 3 shows appetitive tone-signaled data for males and females. The average number of days it took male rats to reach the appetitive criterion (a) was 5.22 ± 0.43 (controls), 5.50 ± 0.64 (restrained), and 6.63 ± 0.82 (recovered). The average number of days it took female rats to reach the appetitive criterion was 6.06 ± 0.55 (controls), 5.17 ± 0.42 (restrained), 4.38 ± 0.32 (recovered). There was no significant effect of sex [*F*_(1,63)_ = 1.478, *p* > 0.05] or condition [*F*_(2,63)_ = 0.156, *p* > 0.05] but there was a significant interaction of sex by condition [*F*_(2,63)_ = 3.319, *p* < 0.05]. Although restraint stress and recovery from restraint incrementally reduced the number of days to the appetitive criterion for females, it had the opposite effect on their male counterparts who needed more days to reach the criterion. There were no significant differences on any pairwise comparisons.

Efficiency ratios (number of correct responses/total number of barpresses) were also measured during acquisition of the first two days of appetitive tone-signaled learning (see Figure 3(b)). For efficiency, we found significant main effects of sex [*F*_(1, 63)_ = 26.131, *p* < 0.01] and condition [*F*_(2,63)_ = 5.237, *p* < 0.01], but also a significant interaction of sex by condition [*F*_(2,63)_ = 6.446, *p* < 0.01]. Pairwise comparisons revealed that control females (0.148 ± 0.004) were slightly more efficient than males (0.121 ± 0.005, *p* = 0.051), but whereas chronic restraint stress improved female efficiency (0.211 ± 0.008) it reduced the male efficiency (0.055 ± 0.002, *p* < 0.05). Recovery had a divergent impact from restraint in that females returned to control-like efficiencies, albeit somewhat lower (0.106 ± 0.009), while males continued to worsen (0.045 ± 0.002, *p* < 0.05).

We also examined latency to barpress during the first two days of acquisition on the appetitive tone-signaled task (Figure 3(c)). We found no significant main effect of sex [*F*_(1,63)_ = 0.963, *p* > 0.05] or condition [*F*_(2,63)_ = 0.216, *p* > 0.05]; however, there was a significant interaction of sex by condition [*F*_(2,63)_ = 3.628, *p* < 0.05]. Whereas restraint stress slowed the barpress response for females with 129.6 ± 6.2 msec (controls) and 141.1 ± 4.2 msec (stressed), males responded more quickly with 151.0 ± 5.4 msec (controls) and 132.2 ± 5.6 msec (stressed). Recovery from restraint had the opposite impact for each sex with females quickening 136.5 ± 4.1 msec and males slowing 138.7 ± 6.7 msec.

### 3.3. Aversive Learning

#### 3.3.1. Escape Shaping

All animals except for one restrained male advanced to aversive learning to begin escape shaping. One index of escape shaping is whether or not they meet criterion. For control animals, 100% of males for both the transfer and AV-only tasks reached criterion, but for females 94% of the transfer animals met criterion and 90% of the AV-only animals met criterion. For restrained animals, 78% of males met criterion for the transfer task whereas 75% met criterion for the AV-only task. In contrast, 100% of females met criterion for both tasks. For the recovery animals, 100% of transfer males met criterion, while 75% of AV-only males met criterion. Finally, 100% of females met both criterions for recovery animals. A chi-square analysis revealed a weak trend for sex differences (*χ* ^2^ = 2.759, *p* = 0.097) but no effect of condition (*χ* ^2^ = 0.845, *p* > 0.05) or task (*χ* ^2^ = 1.077, *p* > 0.05) to meet the escape shaping criterion.

**Figure 4:**
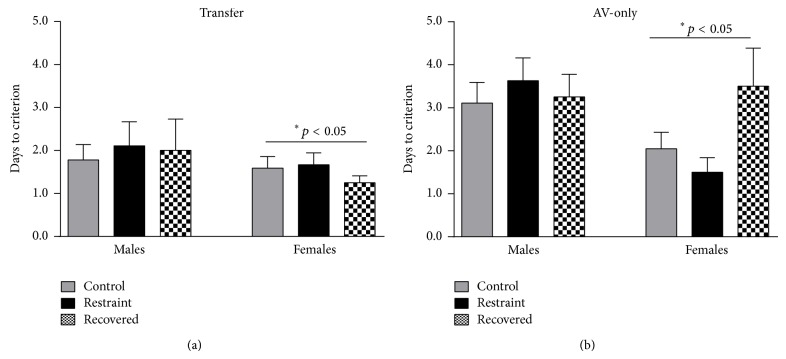
It shows escape shaping for aversive transfer (a) and aversive-only (b) animals for control (gray), stressed (black), and recovered (checkered) animals. There were no significant effects on escape shaping for any transfer animal. There was a main effect of sex for the AV-only animals. Males took longer to reach the escape criterion than females.

Figure 4 shows the average number of days to reach the escape shaping criterion in the aversive paradigm for male and female transfer (a) and AV-only (b) tasks. The average number of days it took the transfer males to reach the escape shaping criterion was 1.78 ± 0.36 (controls, *n* = 9), 2.11 ± 0.56 (restrained, *n* = 9), and 2.0 ± 0.73 (recovered, *n* = 8) and for the aversive-only males was 3.11 ± 0.48 (controls, *n* = 9), 3.63 ± 0.48 (restrained, *n* = 8), and 3.25 ± 0.53 (recovered, *n* = 8). The average number of days it took the transfer females to reach the escape shaping criterion was 1.59 ± 0.27 (controls, *n* = 17), 1.67 ± 0.28 (restrained, *n* = 12), and 1.25 ± 0.16 (recovered, *n* = 8) and for AV-only females was 2.05 ± 0.38 (controls, *n* = 20), 1.50 ± 0.34 (restrained, *n* = 10), and 3.5 ± 0.89 (recovered, *n* = 8). A sex by task by condition analysis revealed significant main effect of sex [*F*_(1,125)_ = 6.904, *p* = 0.01] and a significant main effect of task [*F*_(1,125)_ = 16.321, *p* < 0.01], but not for condition [*F*_(2,125)_ = 0.620, *p* > 0.05]. None of the interaction effects were significant: sex by condition [*F*_(2,125)_ = 1.135, *p* > 0.05], sex by task [*F*_(1,125)_ = 0.891, *p* > 0.05], condition by task [*F*_(2,125)_ = 1.284, *p* > 0.05], or sex by condition by task [*F*_(2,125)_ = 1.878, *p* > 0.05]. Pairwise comparisons showed that females reached the escape shaping criterion more quickly than males did (*p* < 0.05) and transfer animals reached the criterion more quickly than AV-only animals (*p* < 0.05).

#### 3.3.2. Tone-Signaled Learning for Aversive Transfer Animals

**Figure 5:**
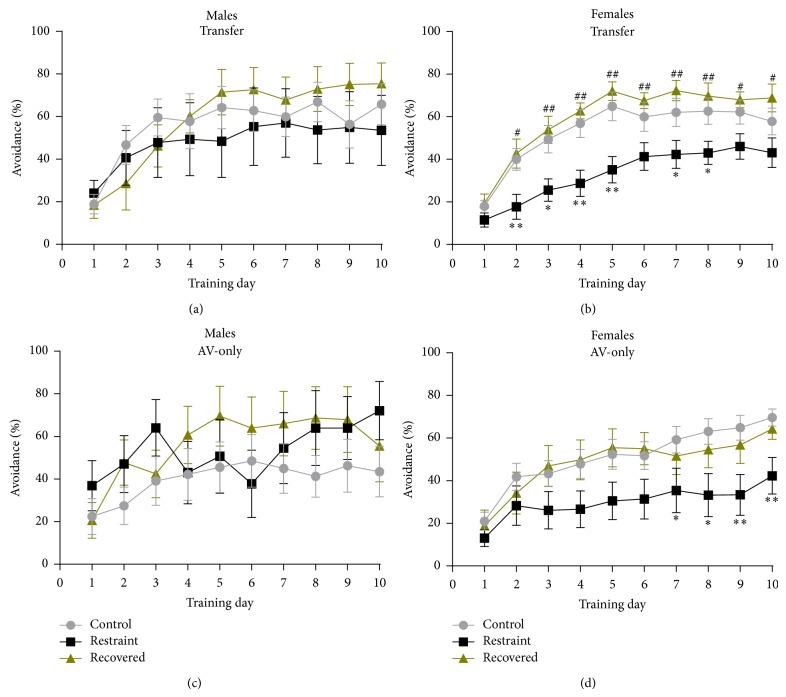
It shows ten-day avoidance rates for transfer males (a) and females (b) and AV-only males (c) and females (d) for control (gray), stressed (black), and recovered (gold) animals. 42-day restraint stress resulted in significant reductions in avoidance rate acquisition and maintenance for transfer females and for maintenance only for AV-only females. However, all recovered animals had avoidance % at rates equal to controls. Day-by-day comparisons for significant differences between control and stressed animals are denoted with ^∗^(*p* < 0.05) or  ^∗∗^(*p* < 0.01), while significant differences between stressed and recovered days are denoted by  ^#^(*p* < 0.05) or  ^##^(*p* < 0.01). Asterisks indicate a significant difference between control and restrained females. There were no significant differences between restrained and recovered females for AV-only.

Animals that successfully reached the escape shaping criterion were advanced to tone-signaled avoidance training.  Figure 5 shows the avoidance % (number  of  correct  responses/total  number  of  trials∗100) for all animals. The average avoidance % for male transfer animals (Figure 5(a)) across the ten-day period was 55.9 ± 9.4% (controls, *n* = 9), 48.5 ± 14.9% (restrained, *n* = 7), and 58.6 ± 8.1% (recovered, *n* = 8). Male transfer animals reached asymptotic performance at an average of day 2.9 ± 0.3 (controls), 3.0 ± 0.9 (restrained), and 5.0 ± 0.3 (recovered). The average avoidance % for female transfer animals (Figure 5(b)) across the ten-day period was 53.4 ± 1.5% (controls), 33.4 ± 1.7% (restrained), and 59.7 ± 5.1% (recovered). The average number of days it took to reach asymptotic performance for female transfer animals was 4.3 ± 0.3 (controls), 4.5 ± 0.5 (restrained), and 4.6 ± 0.2 (recovered). There was a significant effect of learning across time [*F*_(4,226)_ = 57.430, *p* < 0.01] and an interaction of condition by learning across time [*F*_(9,226)_ = 2.276, *p* < 0.01], but there was no significant effect of sex across time [*F*_(4,226)_ = 0.389, *p* > 0.05] nor a significant interaction of sex by condition across time [*F*_(9,226)_ = 0.872, *p* > 0.05]. The data were rerun with independent tests for each sex to examine the effect size of condition. For male rats, there was no effect of condition across time [*F*_(11,114)_ = 1.488, *p* > 0.05] or condition [*F*_(2,20)_ = 0.228, *p* > 0.05], whereas for female rats there was a significant overall effect of condition [*F*_(2,33)_ = 6.487, *p* < 0.01]. Pairwise comparisons found that control females had significantly higher avoidance % than restrained females (*p* < 0.05) and recovered females had significantly higher avoidance % than restrained females (*p* < 0.01), but there was no difference between control and recovered females (*p* > 0.05).

**Figure 6:**
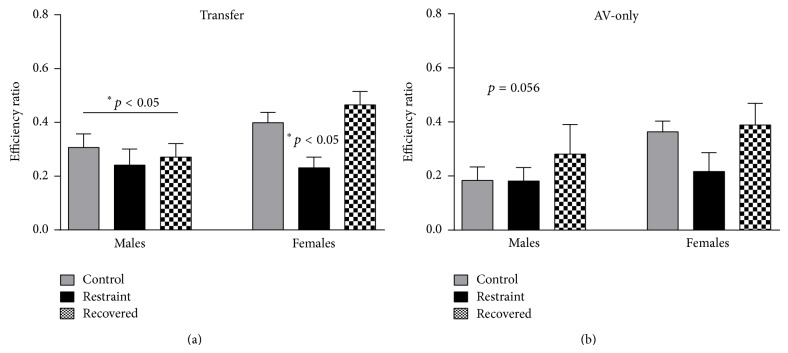
It shows tone-signaled avoidance efficiency ratios (ERs) for transfer (a) and AV-only (b) animals for control (gray), stressed (black), and recovered (checkered) animals. There was a main effect of sex for transfer animals with females avoiding more efficiently. There was also an effect of condition for females with stressed females avoiding significantly less efficiently than control females. A similar pattern was seen for AV-only; however the effect was less robust and showed only a moderate sex effect.

**Figure 7:**
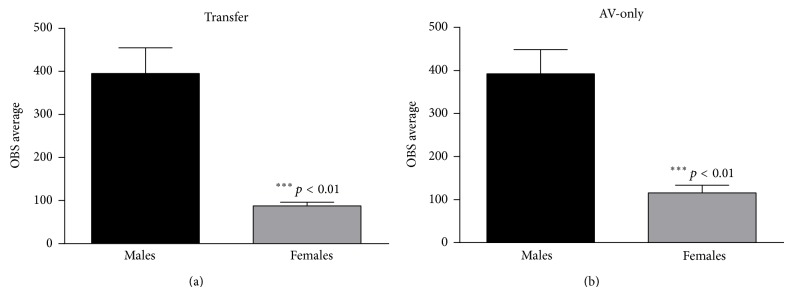
It shows the average number of off-bar shocks (OBSs) across all ten days of avoidance training for transfer (a) and AV-only animals (b). There was an overall main effect of sex for both learning tasks. Males averaged significantly more OBSs.

In addition to analyzing the avoidance percentage, the average avoidance efficiency ratio (number of correct responses/total number of barpresses, ER; see Figure 6(a)) as well as the average number of off-bar shocks (OBSs; Figure 6(b)) was examined. Efficiency ratios (ERs) were averaged across all ten days and there was a main effect of sex [*F*_(1,59)_ = 4.956, *p* < 0.05] and condition [*F*_(2,59)_ = 3.946, *p* < 0.05], but no significant interaction [*F*_(2,59)_ = 1.857, *p* > 0.05]. The average ER for males was 0.307 ± 0.05 (control), 0.241 ± 0.06 (restrained), and 0.271 ± 0.05 (recovered) and for females was 0.399 ± 0.038 (controls), 0.231 ± 0.04 (restrained), and 0.465 ± 0.05 (recovered). In contrast to appetitive learning, restraint stress with recovery impacted male and female ERs in a similar manner, with only the magnitude of change differing between sexes. Finally, OBSs were averaged across the ten-day period which revealed a significant main effect of sex with males averaging 395.4 ± 59.4 and females averaging 87.8 ± 8.6 per session [*F*_(1,59)_ = 34.801, *p* < 0.01; see Figure 7(a)]. There was no main effect of condition [*F*_(2,59)_ = 0.120, *p* > 0.05] or significant interaction of sex by condition [*F*_(2,59)_ = 0.102, *p* > 0.05].

#### 3.3.3. Tone-Signaled Learning for Aversive-Only Animals

Figures 5(c) and 5(d) show avoidance percentages for AV-only animals. The average avoidance % for male AV-only animals (Figure 5(c)) across the ten-day period was 40.1 ± 11.1% (controls, *n* = 9), 53.4 ± 14.9% (restrained, *n* = 6), and 56.3 ± 13.5% (recovered, *n* = 8). The average number of days for AV-only males to reach their asymptote was 3.0 ± 0.6 (controls), 2.8 ± 0.5 (restrained), and 3.8 ± 0.6 (recovered). The average avoidance % for female AV-only animals (Figure 5(d)) across the ten-day period was 51.5 ± 6.0% (controls), 30.0 ± 8.7% (restrained), and 48.7 ± 8.4% (recovered). The average number of days it took each animal to reach asymptotic performance for female AV-only animals was 4.1 ± 0.4 (controls), 3.1 ± 0.6 (restrained), and 4.3 ± 0.5 (recovered). There were a significant effect of learning across time [*F*_(5,265)_ = 23.828, *p* < 0.01] and significant interactions of condition across time [*F*_(10,265)_ = 1.803, *p* < 0.05], as well as sex by condition across time [*F*_(10,265)_ = 1.858, *p* < 0.05]. There was no significant effect for sex across time [*F*_(5,265)_ = 0.590, *p* > 0.05]. When analyzing the sexes separately, there was no significant effect of condition [*F*_(5,18)_ = 0.648, *p* > 0.05] or condition across time [*F*_(12,109)_ = 1.568, *p* > 0.05] for males. For females there was only a trend for condition [*F*_(2,33)_ = 2.997, *p* = 0.064] and no significant effect of condition across time [*F*_(9,146)_ = 1.548, *p* > 0.05].

Efficiency ratios (ERs) and off-bar shocks (OBSs) were also calculated for AV-only animals. The ten-day average ER for male animals was 0.184 ± 0.05 (controls), 0.182 ± 0.05 (restrained), and 0.281 ± 0.11 (recovered) and for female animals was 0.364 ± 0.04 (controls), 0.217 ± 0.07 (restrained), and 0.389 ± 0.08 (recovered). There was a trend for sex differences [*F*_(1,56)_ = 3.836, *p* = 0.056] with males having slightly lower ERs due to nonspecific and higher barpressing, but there was no effect of condition [*F*_(2,56)_ = 1.762, *p* > 0.05] or significant interaction [*F*_(2,56)_ = 0.654, *p* > 0.05] (see Figure 6(b)). A similar and more robust pattern was true for OBSs. Male animals averaged 392.1 ± 56.5 OBSs and female animals averaged 115.6 ± 18.0 for the ten days which was a significant sex difference [*F*_(1,56)_ = 34.491, *p* < 0.01; see Figure 7(b)]. There was no significant effect of condition [*F*_(2,56)_ = 0.591, *p* > 0.05] or significant interaction [*F*_(2,56)_ = 1.584, *p* > 0.05].

#### 3.3.4. Impact of Extended Restraint Duration

We evaluated the impact of the extended duration of restraint on avoidance learning relative to 21-day restraint for transfer and AV-only animals. Daily difference scores were averaged across animals for each restraint condition: (1) 21-day restraint minus control, (2) 42-day restraint minus 21-day restraint, and (3) 42-day restraint minus control. For transfer animals there was a significant main effect of condition [*F*_(2,59)_ = 9.275, *p* < 0.01] and sex [*F*_(1,59)_ = 30.574, *p* < 0.01] and a significant interaction of condition by sex [*F*_(2,59)_ = 4.637, *p* < 0.05]. Post hoc analyses showed significant differences between 21-day restraint minus control and 42-day restraint minus control (*p* < 0.01), as well as a significant difference between 42-day restraint minus 21-day restraint and 42-day restraint minus control (*p* < 0.01), but no significant difference between 21-day restraint minus control and 42-day restraint minus 21-day restraint (*p* > 0.05).

Analyses for AV-only animals showed only an overall trend for condition [*F*_(2,59)_ = 2.605, *p* = 0.083] and a significant main effect of sex [*F*_(1,59)_ = 90.937, *p* < 0.01] and a significant interaction of condition by sex [*F*_(2,59)_ = 10.010, *p* < 0.01]. However, post hoc analyses showed no significant differences between any conditions, 21-day restraint minus control versus 42-day restraint minus control (*p* > 0.05) and 42-day restraint minus 21-day restraint versus 42-day restraint minus control (*p* > 0.05), nor between 21-day restraint minus control and 42-day restraint minus 21-day restraint (*p* > 0.05).

## 4. Discussion

The current paper is a follow-up to our manuscript published last year [18] where we reported sex differences on the impact of three weeks of chronic restraint stress on an appetitive-to-aversive instrumental learning task. The current study extended the duration of chronic restraint stress to six weeks, which has been used by others [23]. Secondly, the present study tested the ability of the animals to recover from six weeks of chronic restraint stress following a postrestraint period with and without an intervening 21-day recovery period prior to testing. The current study found multiple effects of sex, condition, and task, as well as significant interactions.

### 4.1. Physiological Impact of Chronic Stress

In our previous publication, we indexed the physiological impact of the commonly used 21-day chronic restraint stress model with daily body weight measurement, which has been previously utilized as an index of stress [22]. The data presented for body weight assessment are from the same set of littermate animals that we published the first 21 days of restraint for [18]. Here we show days 22 through 42 to demonstrate weight gain across conditions. Similar to their first 21 days, the restrained males continued to gain weight at an attenuated rate relative to their control counterparts. In contrast, although restrained females remained somewhat smaller they did not continue to lose weight relative to their control littermates. Although the impact of chronic stress is progressive, reports of habituation for any one systemic measure have been previously noted [24]. Thus, the current results shed light on the divergent time-course of the impact of chronic restraint on male and female rats. In light of the learning deficits found, this habituation underscores the notion of multiple systems being impacted at different times and ultimately leading to sexually dimorphic and task specific outcomes.

### 4.2. Appetitive Learning

#### 4.2.1. Appetitive Shaping

The two primary findings from the appetitive shaping task found a significant interaction of sex by condition on the FR-1 task and a main effect of sex on the FR-4 task. In both tasks, females took longer to reach the shaping criterions suggesting that the males were either more motivated to barpress for food reward or simply faster at it which matches previous reports in the literature [25, 26]. A new finding was the impact of condition on the appetitive training. Previously we found that 21 days of chronic restraint stress facilitated appetitive learning in females [18], but we did not see that with six weeks of chronic restraint stress. However, we did see an increase in the number of days to meet criterion during FR-1 for the recovered females. The attenuation of the appetitive facilitation along with the effect in recovered females suggests that there may be a compensatory response to chronic restraint stress that occurs from day 22 to day 42, which ultimately leads to an even slower response for recovered females. It is possible that the females progressed from heightened arousal responsiveness to the appetitive stimulus during the initial 21 days to a responsiveness characterized by anhedonia, a behavior indicating a lack of interest which frequently accompanies a mood disorder [27, 28].

#### 4.2.2. Appetitive Tone-Signaled Learning

The data show multiple effects on appetitive tone-signaled learning. Although the impact of restraint on females still enhanced their performance similar to that previously seen in 21-day restrained females relative to control females, the effect was not significant for 42-day restrained females again suggesting the onset of anhedonia and an impact on striatal circuitry. However, we did find significant sex by condition interactions for the number of days to meet criterion, for efficiency ratios during acquisition, and for the latency to barpress during acquisition. Females were overall more efficient than males during acquisition which matches our previous report [18]. Males that were chronically stressed needed more time to reach criterion and were less efficient than control males whereas chronic stress had the opposite impact on females. In contrast, the opposite pattern of results was found for latency to barpress during acquisition. Chronic stress sped up male responses while it slowed female responses suggesting a sexually divergent response speed. In all likelihood the faster responses seen in males may have precluded sufficient information processing leading to a less efficient response. This pattern of results suggests that males may be biologically disadvantaged in expressing behavioral inhibition specifically for appetitive food reward, which one study has suggested is modulated by serotonergic differences [29]. Alternatively, the male rats may be experiencing overall deficits in behavioral inhibition indicating deficits in prefrontal cortical control over stimulus-response circuitry. Regardless, it is clear that chronic stress is exacerbating the response.

### 4.3. Aversive Learning

#### 4.3.1. Aversive Escape Shaping

In contrast to 21 days of restraint stress [18], we found no significant effects during escape shaping for any transfer animal, but chronic stress did have an impact on escape shaping for AV-only animals. Previously we found that chronically stressed females were facilitated relative to control females in transfer aversive learning. Similar to the appetitive data, the 42-day restrained females are no longer facilitated further supporting the notion that the heightened arousal 21-day females were experiencing changes into a depressive and unmotivated state. These results suggest that the 42-day stressed females were experiencing an overall deficit in motivation rather than a valence-specific deficit.

Similar to our previous findings [18], we found a significant main effect of task where AV-only animals took longer to meet the escape shaping criterion than animals with prior appetitive training. Also, we saw a main effect of sex on the AV-only task where female animals met criterion significantly faster than males with the exception being the recovered females who met criterion at a comparable rate to males. Interestingly, the recovered female AV-only animals shaped more slowly than control or restrained females which matches the pattern found in the FR-1 shaping task and suggests that even though the chronic restraint group was not significantly different from control animals on escape shaping, there was nonetheless a meaningful impact. It is possible that multiple systems were impacted at different times or alternatively that the impact of chronic stress on the same system when extended beyond three weeks does not have a corresponding linear impact on function.

#### 4.3.2. Aversive Tone-Signaled Learning

Following advancement to tone-signaled learning, the 42-day restrained transfer animals showed exacerbated deficits relative to those previously seen with 21-day restraint [18]. The 42-day restrained transfer females showed deficits in avoidance % during learning acquisition, transition, and maintenance, which provides further support of an overall deficit in motivation and a disruption in reward circuitry (for review see [28]). In contrast, all males continued to be highly responsiveness and show increased variability in their responding. The 42-day restrained males showed a similar pattern as reported in 21-day restrained males and as seen in the appetitive task of high responsiveness and low efficiency suggesting that their primary issue may have been decreased behavioral inhibition due to heightened arousal.

Relative to the 21-day restrained animals, different patterns emerged on the AV-only task for both males and females. For males, there was increased variability in barpressing which was the highest during the AV-only task. This variability was likely driven by the overall increase in off-bar shocks (OBSs) during AV-only relative to aversive transfer. There was little impact of chronic restraint on overall avoidance % for males, but there was an effect on response efficiency. Males continued to show overall high but inefficient levels of responsiveness with the impact of restraint being an exacerbation on inefficiency. Given that restrained males were significantly faster responders it is likely that they were experiencing heightened arousal which resulted in proactive interference. Males had significantly higher numbers of OBSs alongside their nonefficient but robust response pattern which lead to an overall increase in response variability within animal on a single session as well as across sessions especially on the AV-only task. The current set of results suggests that on the AV-only task the males' change in responsiveness is more drastic representing an overall shift in performance strategy. On days 4 through 6, restrained males reduced their average number of barpresses, but their ERs did not improve indicating that they did not have an increased understanding of the task demands but rather they suffered “response maintenance fatigue.” It is not clear if this fatigue was related to a physical demand overload or a cognitive strain or potentially some combination.

In contrast, females appeared to suffer from an overall decreased motivation which impacted their avoidance rates on the AV-only task as well. However, the magnitude of the deficit was smaller relative to the transfer task and existed only on postasymptotic training days relative to the larger transfer deficit which was also present during learning acquisition and transition. The combined results from the appetitive, transfer, and AV-only tasks suggest that 22 to 42 days of chronic restraint stress may be a sensitive time period for impacting motivation for females and that higher cognitive tasks may be the most vulnerable to these effects, but not exclusively so.

#### 4.3.3. Contribution of the Extended Restraint Duration

The impact of the extended restraint revealed a dose-dependent response on female transfer learning. After analyzing difference scores between groups, we saw a significant effect for 21-day restraint minus control versus 42-day restraint minus control which suggest that the impact of each of these durations is significantly different from each other with the 42-day restraint minus control having the larger impact. However, when directly comparing the effect sizes for the first three weeks versus the final three weeks (21-day restraint minus control versus 42-day restraint minus 21-day restraint) there was no significant difference. This suggests that while the impact of chronic restraint stress is progressive, the added contribution of the final three weeks of restraint did not substantially differ from the initial three weeks.

### 4.4. Recovery from Extended Restraint Stress

There is little research investigating recovery from the impact of 42 days of restraint stress. The concept of recovery encapsulates the idea of undoing harm such that system homeostasis is reestablished [30]. Yet experience and time are progressive and ongoing which questions the idea of a true reversal of a harmful experience, but rather a successful adaptation is achieved. Thus, recovery may not simply be the “undoing” of the impact but is a dynamic and forward process that integrates all new information with the old. For the most part our results suggest that recovery from 42-day chronic restraint is a dynamic forward process, but the results from the avoidance % data indicate a complete reversal of the deficit for females. It may be that different systems recover at different rates and that with additional time a complete reversal would emerge on all tasks. Alternatively, there may be activational differences in system recovery processes.

There are a few limitations of this study to mention. First, although 21-day chronic restraint stress has been well characterized, we did not specifically assess the neural correlates underlying the behavioral effects seen in the paradigm. Secondly, in addition to gonadal hormones moderating the effects of chronic stress on learning, menstrual phase has been shown to do this as well [31–33]. Since our learning paradigms extend across weeks, the impact of chronic stress likely represents the summation across multiple menstrual cycles. However, we did not assess the menstrual phase of our females so we do not know if they were cycling normally.

## 5. Conclusion

The primary impact of chronic restraint stress revealed heightened responsiveness for males leading to further response variability and inefficient responding which interfered with task demands. Although we also saw high variability in male avoidance rates with 21-day restraint, we did not see an effect on response speed until 42-day restraint indicating a progressive impact on males. For stressed females, there was a decrease in responding and in response speed, but no decrease in response efficiency indicating an overall pattern of decreased motivation which was reversible. Given that both sexes are experiencing performance deficits, albeit opposing ones, one utility of this study was to shed light on areas that can be targeted for effective learning intervention techniques which vary according to sex, stressor duration, and task demands. Finally, it is important to note that, even with an extended stressor duration, animals were able to recover from the avoidance deficits.
